# Influence of ^60^Co-γ Irradiation on the Components of Essential Oil of Curcuma

**DOI:** 10.3390/molecules28155877

**Published:** 2023-08-04

**Authors:** Chang Lei, Jianjun Liu, Wenchao Zhou, Wei Zhou, Shunxiang Li, Dan Huang

**Affiliations:** 1 State Key Laboratory of Chinese Medicine Powder and Medicine Innovation in Hunan (Incubation), Science and Technology Innovation Center, Hunan University of Chinese Medicine, Changsha 410208, China; leichang@hnucm.edu.cn (C.L.); liujianjun201808@163.com (J.L.); zhouwenchao202307@163.com (W.Z.); fxxx99w@163.com (W.Z.); 2 Hunan Engineering Technology Research Center for Bioactive Substance Discovery of Chinese Medicine, School of Pharmacy, Hunan University of Chinese Medicine, Changsha 410208, China; 3 Hunan Province Sino-US International Joint Research Center for Therapeutic Drugs of Senile Degenerative Diseases, Changsha 410208, China

**Keywords:** Curcuma, essential oils, chemical compounds, ^60^Co irradiation, GC-IMS

## Abstract

The gas chromatography–ion mobility spectrometry (GC-IMS) method is a new technology for detecting volatile organic compounds. This study was carried out to evaluate the effects of volatile aroma compounds of Curcuma essential oils (EOs) after ^60^Co radiation by GC-IMS. Dosages of 0, 5, and 10 kGy of ^60^Co were used to analyze EOs of Curcuma after ^60^Co irradiation (named EZ-1, EZ-2, and EZ-3). The odor fingerprints of volatile organic compounds in different EOs of Curcuma samples were constructed by headspace solid-phase microextraction and GC-IMS after irradiation. The differences in odor fingerprints of EOs were compared by principal component analysis (PCA). A total of 92 compounds were detected and 65 compounds were identified, most of which were ketones, aldehydes, esters, and a small portion were furan compounds. It was found that the volatile matter content of 0 kGy and 5 kGy was closer, and the use of 10 kGy ^60^Co irradiation would have an unstable effect on the EOs. In summary, it is not advisable to use a higher dose when using ^60^Co irradiation for sterilization of Curcuma. Due to the small gradient of irradiation dose used in the experiment, the irradiation dose can be adjusted appropriately according to the required sterilization requirements during the production and storage process of Curcuma to obtain the best irradiation conditions. GC-IMS has the advantages of GC’s high separation capability and IMS’s fast response, high resolution, and high sensitivity, and the sample requires almost no pretreatment; it can be widely used in the analysis of traditional Chinese medicines containing volatile components. It is shown that irradiation technology has good application prospects in the sterilization of traditional Chinese medicines, but the changes in irradiation dose and chemical composition must be paid attention to.

## 1. Introduction

Curcuma is widely used in traditional Chinese medicine. It is often used to promote the flow of qi, eliminate blood stasis with strong effect, and relieve pain by removing the stagnation of undigested food [1]. The pharmacological effects of essential oils (EOs) in Curcuma mainly include anti-tumor, anti-inflammatory analgesic, and antiviral effects, as well as protection of the cerebral tube, influence on the nervous system, protection of the kidneys, antioxidation, termination of pregnancy and prevention of early pregnancy, liver protection, cytotoxicity effects, antidepressant effects, and anti-diabetic effects [2,3,4,5]. In particular, the anti-tumor effect has attracted the attention of many researchers and has been widely used in clinical practice. It can be seen that Curcuma, as a commonly used clinical traditional Chinese medicine in the ginger family, has great medicinal value and is worthy of our in-depth development and utilization. 

An efficient and rapid sterilization method, ^60^Co irradiation is the use of high-energy rays produced by γ-ray ionizing radiation to produce powerful physical and biological effects in the process of energy transfer to achieve insecticidal effect, sterilization, and inhibition of physiological processes. The principle is mainly to destroy the DNA and RNA in microbial cells so that the damaged DNA and RNA are degraded, and the synthetic protein and genetic functions are lost to achieve the effect of killing cells [6]. Chinese medicine is highly susceptible to microbial contamination because of its complex composition. ^60^Co irradiation sterilization has become more popular in the traditional pharmaceutical industry because it has the characteristics of cold treatment, strong penetration, simple operation, and continuous operation [7]. However, some traditional Chinese medicines have changed their chemical composition after irradiation, such as Gentianae macrophyllae Radix, Gentianae Radix et Rhizoma, Asteris Radix et Rhizoma, Bambusae concretio Silicea and Physalis calyx seu Fructus.

The GC-IMS method is a new technology for detecting volatile organic compounds. Here, the substances are further separated in the IMS drift tube. Analysis of IMS as a detector for GC can achieve two-dimensional separation of volatile organic substances [8]. It has the advantages of GC’s high separation capability and IMS’s fast response, high resolution, and high sensitivity, and the sample requires almost no pretreatment [9]. It is used in the analysis of foods containing volatile components.

In previous studies, the volatile oil of turmeric was extracted by steam distillation and irradiated with ^60^Co rays of different intensities (dosages of 0, 5, and 10 kGy). The results showed that 97 volatile components were detected in turmeric volatile oil and 64 components were identified by database retrieval. With the change in irradiation intensity, the volatile components in the three turmeric volatile oil samples were similar but there were significant differences in peak intensities. In general, different doses of ^60^Co irradiation can affect the content of volatile substances in turmeric volatile oil. As the irradiation dose increases, the peak area decreases, so the best irradiation dose is 5 kGy/min.

The essential oil of Curcuma, as its bioactive ingredient, is rich in chemical constituents. However, there are almost no reports in the literature of a study of the chemical constituents of Curcuma essential oil after ^60^Co radiation by GC-IMS. We describe here for the first time the chemical components of the essential oils of Curcuma by GC-IMS. The purpose of this work is to determine the changes in essential oil composition under different irradiation intensities of ^60^Co and to select an appropriate irradiation dose using GC-IMS.

## 2. Results

### 2.1. GC-IMS Analysis of EOs in Curcuma

**Figure 1:**
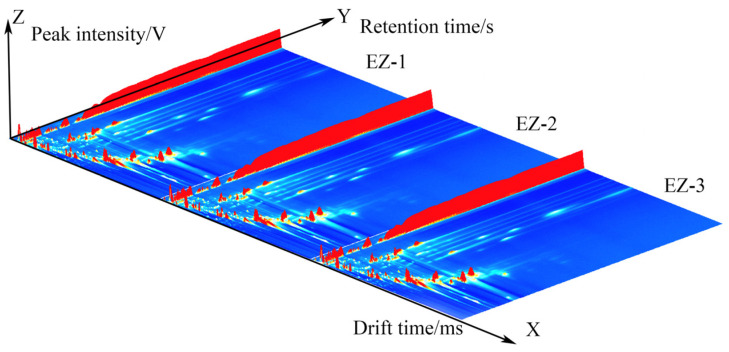
A comparison of the GC-IMS 3D spectra.

The data generated by the instrument constitute a three-dimensional spectrogram (retention time, migration time, and peak intensity), as shown in Figure 1, from which the differences in volatile organic compounds in different samples can be intuitively seen. However, because of the inconvenience of observation, the following top view is taken for comparison of differences.

**Figure 2:**
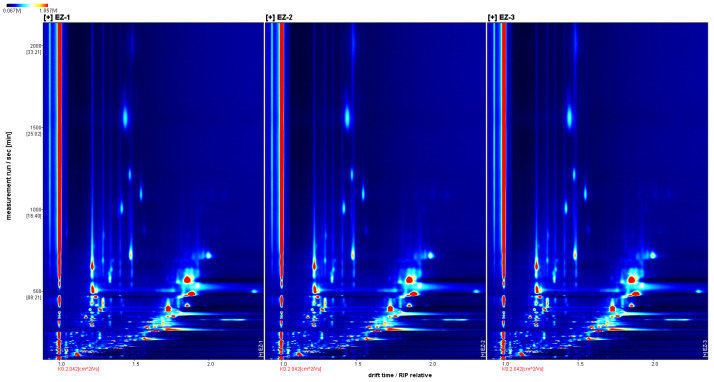
GC-IMS chromatograms of samples.

A two-dimensional top view of the compounds in Curcuma essential oils was generated using the Reporter plug-in, as shown in Figure 2. It consists of drift time, retention time, and ion signal intensity. The background of the whole figure is blue and the red vertical line at the abscissa at 1.0 is the RIP (reactive ion peak, normalized). The ordinate coordinate represents the retention time(s) of gas chromatography and the abscissa represents the ion migration time (normalization process). Each point on either side of the RIP represents a volatile organic compound. The color represents the concentration of the substance; white indicates a lower concentration, red indicates a higher concentration, and a darker color indicates a greater concentration.

**Figure 3:**
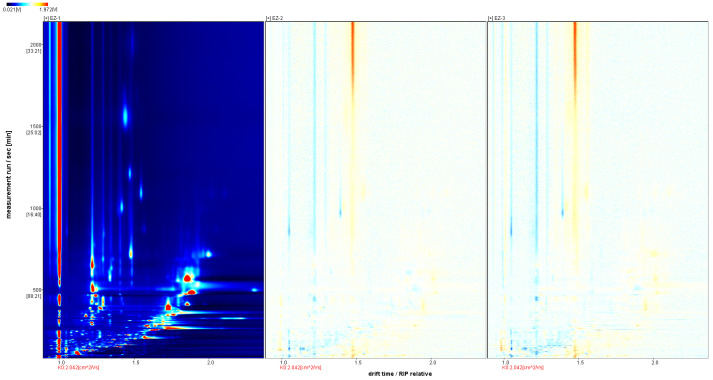
Difference analysis of GC-IMS of samples.

We use the Reporter plug-in to obtain a GC-IMS difference plot of the sample, as shown in Figure 3. We use EZ-1 as a reference and the rest of the spectra to subtract the signal peaks in EZ-1 to obtain a difference spectrum of the two. Regions with fewer points indicate that the substance is lower than EZ-1 in this sample and regions with more points indicate that the substance is more than EZ-1 in this sample. Similarly, the darker is the color, the greater is the difference.

### 2.2. EO Fingerprint Comparison of Samples

**Figure 4:**
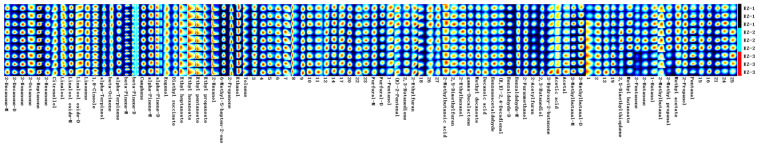
Gallery Plot of selected essential oil compounds by GC-IMS.

To visually and quantitatively compare EO differences between different samples, using the Gallery Plot plug-in, we obtain a Gallery Plot of the sample, as shown in Figure 4. Each row in the figure represents all the signal peaks selected in a sample. Each column in the figure represents the signal peak of the same volatile organic compound in different samples. Some of the substances are followed by _M and _D, which are monomers and dimers of the same substance, and the numbers are unidentified peaks. The complete EO information for each sample and the differences between EOs between samples can be seen from Figure 4.

### 2.3. PCA of EOs in Samples

**Figure 5:**
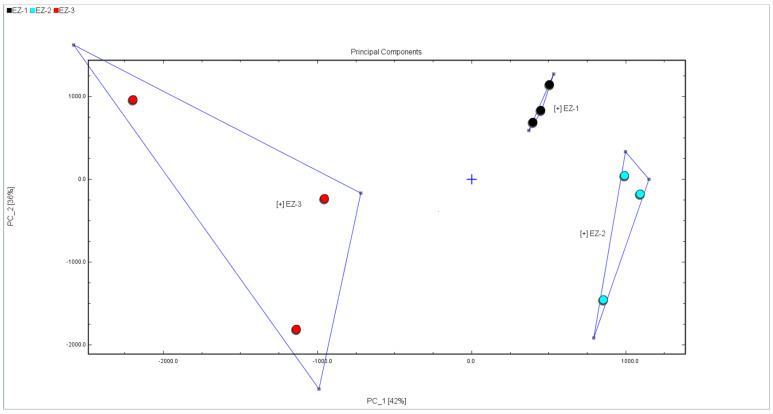
PCA analysis plot of the sample.

**Figure 6:**
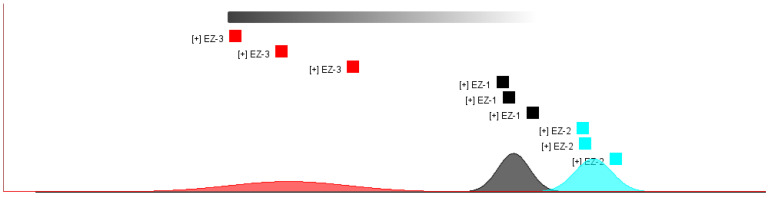
Sample’s nearest neighbor–Euclidean distance map. (The closer the distance, the higher the similarity.)

PCA is a multivariate data analysis tool for analyzing cubes with quantitative variables [10]. By using several main component factors to represent many other complex and hard-to-find variables in the original sample, we compare the differences between different samples. PCA of Curcuma essential oils was performed, and the results are shown in Figure 5 and Figure 6. It can be seen from the figure that the composition of the essential oil after the treatment of the three methods is not the same, among which EZ-1 and EZ-2 are slightly more similar and EZ-3 is quite different from the other samples.

### 2.4. Chemical Composition of the EOs

Using the NIST database and IMS database built into VOCal software(Version 0.4.03, GAS Deutschland, Dortmund, Germany) and other plugins (Reporter plugin, Gallery Plot plugin, Dynamic PCA plugin), we can qualitatively analyze the substance. A total of 65 chemical components have been identified from Curcuma essential oils after ^60^Co irradiation and the composition identification results are shown in Table 1. Because of the higher dimer content of compounds such as 2-decanone, linalool oxide, and beta-pinene, two peaks were present; these correspond to monomers and dimers. As can be seen from Table 1 and Table 2, the main chemical components of Curcuma essential oil include esters, aldehydes, terpenes, alcohols, ketones, and acids. 

## 3. Discussion

This study used GC-IMS to analyze the essential oils of Curcuma under different doses of irradiation. In the obtained three-dimensional, two-dimensional, and differential comparison spectra, it was clearly observed that there were differences in the content of volatile components among the three irradiation dose components. To confirm the conclusion, a PCA differential analysis model was established and the Euclidean distance between samples was calculated to reduce the dimensionality of the data for visualization [11,12]. It can be more intuitively understood that the volatile components of EZ-1 and EZ-2 samples are closer, while EZ-3 samples have significant differences from the other two components when they are far apart, and the three samples between groups are also relatively dispersed; the dosage of 10 kGy has a significant and unstable impact on the volatile component content of the essential oil from Curcuma. Qualitative analysis was conducted on the volatile components in the essential oil of Curcuma and a total of 92 compounds were detected. Among them, 65 compounds were able to be identified and 27 compounds were temporarily unable to be confirmed due to incomplete databases. The confirmed compounds include 11 ketones, 10 aldehydes, 9 esters, 6 terpenoids, and a small amount of acid compounds. 

It can be seen from the fingerprint that the contents of furfural, 1-pentanol, E-2-pentenal, 2,3-butanedione, 2-ethylfuran, and other substances are higher in EZ-1. The contents of methyl 2,5-dimethylthiophene, methyl butyrate, 2-pentanone, 2-butanone, 1-butanol, 2-methylbutyral, methyl acetate, 2-propanol, valeraldehyde, and other substances in EZ-2 are higher. The contents of γ-caprolactone, methyl caprate, capric acid, phenylacetaldehyde, (E, E)-2,4-decadienoal, benzaldehyde, 2-furanmethanol, 2-acetylfuran, 2,3-butanediol, 3-hydroxy-2-butanone, acetic acid, acetal, and other substances in EZ-3 are higher. If there are specific requirements for the selection of the content of a certain component, the appropriate irradiation dosage is recommended.

This is somewhat different from the volatile oil of Curcuma detected using GC-MS technology [13,14,15,16]. The possible reason is that more small molecule substances are detected. 

Modern pharmacology has shown that Curcuma has anti-tumor effects, anti-inflammatory effects, and anticoagulant effects, as well as improving liver and kidney function [17,18,19,20,21,22,23]. It has very high medicinal value, and most of the component analysis of Curcuma is carried out using GC-MS technology. This study uses GC-IMS technology to analyze the volatile oil of Curcuma, which can better detect substances with lower thresholds, it can provide a more detailed report for the pharmacological study of effective substances in the volatile oil of Curcuma. At the same time, it also provides a certain selection basis for the dosage standard of ^60^Co radiation sterilization used in the production and storage of traditional Chinese medicinal materials of Curcuma, which has high research significance.

^60^Co irradiation is a high-energy ray produced by γ-ray ionizing radiation, which is used for efficient and rapid sterilization. The principle is to destroy DNA and RNA in microbial cells, thereby killing cells. The source of traditional Chinese medicine is complex and it is extremely polluted by microorganisms and some harmful pests. When other sterilization methods such as high temperature destroy the ingredients of traditional Chinese medicine, irradiation sterilization becomes a good method, and it is easy to operate and has strong penetrating power. But some traditional Chinese medicines have changed their chemical composition after irradiation, such as Gentianae macrophyllae Radix, Gentianae Radix et Rhizoma, Asteris Radix et Rhizoma, and Physalis calyx seu Fructus. So, the changes in irradiation dose and chemical composition must be paid attention to.

## 4. Materials and Methods

### 4.1. Plant Material

Curcuma (the rhizome of *Curcuma wenyujin* Y. H. Chen et C. Ling) was gathered from Baise, China and identified by Prof. Zhaoming Xie at the Hunan Academy of Traditional Chinese Medicine. A voucher specimen (HNATCM2021-012) was deposited in the herbarium of the Hunan Academy of Traditional Chinese Medicine.

### 4.2. Isolation of the EOs

The sample (50 g) was subjected to hydro-distillation in a Clevenger-type apparatus for 5 h in accordance with the Pharmacopoeia of China (2020). In brief, a sample was added to 300 mL of distilled deionized water in a 1.0 L round-bottomed flask and heated to boiling, after which the essential oil was evaporated together with water vapor and finally collected in a condenser. The essential oil layer was separated, preserved in a sealed sample tube, and then stored in the dark (away from light) at 4 °C for analysis. The yield of extraction was 2.2% (*w*/*w*) based on the weight of sample.

### 4.3. ^60^Co Irradiation

The resulting Curcuma EOs were dehydrated with anhydrous Na_2_SO_4_ and then divided into three equal parts for ^60^Co irradiation (store in 1.5 mL sealed vials), radiation source intensity 2.96 × 10^16^ Bq, irradiation method: dynamic stepping. Dose rates were 0, 5, and 10 kGy/min; irradiation time was 6 h. The ^60^Co γ radiation source (^60^Co class I radioactive source irradiation equipment, Huangshi, China) was located at Hunan Radiological Technology Application Research Center (Changsha, China). 

### 4.4. GC-IMS Analysis

The GC-IMS analysis was performed using GC coupled with an ion mobility spectrometry instrument (Flavourspec^®^-G.A.S., Dortmund, Germany). The sample enters the instrument with the carrier gas, first through the initial separation of the gas chromatography column and then into the ion migration tube. After the ionization of the molecule to be measured, under the action of the electric field and the reverse drift gas, it migrates to the Faraday disc for detection to achieve secondary separation. 

We take 50 μL of sample, load it into a 20 mL headspace flask, heat the headspace vial at 80 °C for 10 min, and incubate it at 500 rpm. Automatic headspace injection volume is 100 μL and the temperature of the injection needle is 85 °C for headspace injection analysis. 

GC conditions: The column is an MXT-5 column (15 m × 0.53 mm × 1 μm), the column temperature is 60 °C, and the carrier gas is N_2_. The carrier air velocity program is initially 2.0 mL/min, which is held for 2 min, linearly increased to 100.0 mL/min at 2 min to 20 min, and maintained at a flow rate of 100.0 mL/min for 20 min to 40 min. Flow is then stopped for a total running time of 40 min.

IMS conditions: drift gas is N_2_ and drift gas velocity is 150 mL/min.

### 4.5. Statistical Analysis

Using the NIST 17 database, we identified compounds in the GC-IMS data by comparing the linear retention indices and mass spectra. The GC-IMS data were examined using the special software including LAV (from G.A.S., Dortmund, Germany version 2.0.0), Reporter, Gallery Plot, and GC-IMS Library Search. Using NIST Library and IMS database retrieval software from G.A.S., we determined the detected EOs by combining the retention index (RI) and drift time (Dt).

## 5. Conclusions

The experimental results showed that irradiation with 0, 5, and 10 kGy on the volatile oil of Curcuma does not produce new substances, but it can change the content of its volatile substances and the effects on different substances are inconsistent. Using GC-IMS for qualitative analysis of volatile compounds, a total of 92 compounds were detected, most of which were ketones, aldehydes, and esters, and a small portion were furan compounds. GC-IMS detection has more efficient separation ability and sensitive response speed, and can detect small molecule compounds without sample pretreatment, making it more effective for analyzing the differences in volatile components among different samples. By analyzing the GC-IMS fingerprint, PCA, and adjacent Euclidean distance map of the sample, it was found that the volatile matter content of 0 kGy and 5 kGy was closer, and the use of 10 kGy ^60^Co irradiation would have an unstable effect on the EOs. In summary, it is not advisable to use a higher dose when using ^60^Co irradiation for sterilization of Curcuma. Due to the small gradient of irradiation dose used in the experiment, the irradiation dose can be adjusted appropriately according to the required sterilization requirements during the production and storage process of Curcuma to obtain the best irradiation conditions. The results indicated that irradiation has a certain effect on the composition of Curcuma EOs; with the increase in irradiation dose, some composition of Curcuma EOs changed. This study provides a sound basis for the use of ^60^Co-γ ray irradiation sterilization technology during the preparation of medicinal herbs for the effective destruction of mycotoxin contamination. 

It shows that irradiation technology has a good application prospect in the sterilization of foods and traditional Chinese medicine containing volatile components, but the changes in irradiation dose and chemical composition must be paid attention to. GC-IMS has the advantages of simple operation, strong separation ability, short detection cycle, and the ability to preserve the original taste of samples to the greatest extent, and can be successfully applied to foods and traditional Chinese medicine.

## Figures and Tables

**Table 1:** Results of component analysis of Curcuma essential oils.

Count	Compound Name	CAS	RI	Molecular Formula	Rt/s	Dt/ms (RIPrel)	Comment
1	gamma-Decalactone	C706149	1598.5	C_10_H_18_O_2_	1209.228	1.47454	-
2	Methyl decanoate	C110429	1508.0	C_11_H_22_O_2_	1079.39	1.54878	-
3	(E,E)-2,4-Decadienal	C25152845	1454.2	C_10_H_16_O	1002.04	1.42077	-
4	Decanoic acid	C334485	1360.2	C_10_H_20_O_2_	867.201	1.57124	-
5	Eugenol	C97530	1341.6	C_10_H_12_O_2_	840.393	1.29711	-
6	2-Decanone	C693549	1256.7	C_10_H_20_O	718.508	1.48226	Monomers
7	2-Decanone	C693549	1255.1	C_10_H_20_O	716.197	1.99783	Dimers
8	alpha-Terpineol	C98555	1206.8	C_10_H_18_O	646.87	1.22248	-
9	Diethyl succinate	C123251	1234.7	C_8_H_14_O_4_	686.937	1.29422	-
10	Citronellol	C106229	1197.5	C_10_H_20_O	633.591	1.34963	-
11	Linalool	C78706	1106.9	C_10_H_18_O	503.438	1.2229	-
12	Ethyl heptanoate	C106309	1122.5	C_9_H_18_O_2_	525.872	1.9311	-
13	2-Nonanone	C821556	1091.9	C_9_H_18_O	481.9	1.88944	-
14	Linalool oxide	C60047178	1080.6	C_10_H_18_O_2_	465.66	1.24616	Monomers
15	Linalool oxide	C60047178	1081.0	C_10_H_18_O_2_	466.245	1.8138	Dimers
16	1,8-Cineole	C470826	1027.6	C_10_H_18_O	389.61	1.73145	-
17	beta-Ocimene	C13877913	1054.5	C_10_H_16_	428.22	1.21822	-
18	Benzeneacetaldehyde	C122781	1045.1	C_8_H_8_O	414.765	1.2491	-
19	Limonene	C138863	1025.6	C_10_H_16_	386.685	1.20792	-
20	alpha-Terpinene	C99865	1015.0	C_10_H_16_	371.475	1.22263	-
21	6-Methyl-5-hepten-2-one	C110930	990.5	C_8_H_14_O	339.885	1.17704	-
22	beta-Pinene	C127913	976.0	C_10_H_16_	327.6	1.21969	Monomers
23	beta-Pinene	C127913	976.0	C_10_H_16_	327.6	1.64175	Dimers
24	Camphene	C79925	944.9	C_10_H_16_	301.275	1.21822	-
25	alpha-Pinene	C80568	931.8	C_10_H_16_	290.16	1.22116	Monomers
26	alpha-Pinene	C80568	931.1	C_10_H_16_	289.575	1.67557	Dimers
27	2-Ethylhexanol	C104767	1018.3	C_8_H_18_O	376.21	1.79856	-
28	Ethyl hexanoate	C123660	1007.5	C_8_H_16_O_2_	360.735	1.81595	-
29	2-Octanone	C111137	999.2	C_8_H_16_O	348.832	1.7651	-
30	Benzaldehyde	C100527	959.8	C_7_H_6_O	313.914	1.15225	Monomers
31	Benzaldehyde	C100527	959.4	C_7_H_6_O	313.517	1.47206	Dimers
32	Ethyl pentanoate	C539822	901.2	C_7_H_14_O_2_	264.315	1.71158	-
33	2-Heptanone	C110430	891.6	C_7_H_14_O	256.379	1.62862	-
34	2-Furanmethanol	C98000	873.3	C_5_H_6_O_2_	246.856	1.1188	-
35	2-Acetylfuran	C1192627	911.1	C_6_H_6_O_2_	272.648	1.45333	-
36	2,5-Dimethylthiophene	C638028	856.5	C_6_H_8_S	238.127	1.07866	-
37	Furfural	C98011	828.3	C_5_H_4_O_2_	223.446	1.08401	Monomers
38	Furfural	C98011	826.7	C_5_H_4_O_2_	222.652	1.3329	Dimers
39	2-Methylbutanoic acid	C116530	827.5	C_5_H_10_O_2_	223.049	1.46938	-
40	2-Hexanol	C626937	793.1	C_6_H_14_O	205.193	1.56974	-
41	2-Hexanone	C591786	791.9	C_6_H_12_O	204.546	1.50912	-
42	2,3-Butanediol	C513859	778.3	C_4_H_10_O_2_	198.14	1.3627	-
43	1-Pentanol	C71410	760.5	C_5_H_12_O	190.908	1.25055	-
44	Acetal	C105577	748.9	C_6_H_14_O_2_	186.155	0.96913	-
45	(E)-2-Pentenal	C1576870	747.3	C_5_H_8_O	185.535	1.36062	-
46	3-Hydroxy-2-butanone	C513860	733.1	C_4_H_8_O_2_	179.749	1.33778	-
47	2,5-Dimethylfuran	C625865	739.9	C_6_H_8_O	182.503	1.0218	-
48	Methyl butanoate	C623427	716.5	C_5_H_10_O_2_	172.993	1.15331	-
49	Ethyl propanoate	C105373	695.4	C_5_H_10_O_2_	164.398	1.44128	-
50	2-Pentanone	C107879	686.6	C_5_H_10_O	161.106	1.37552	-
51	2-Ethylfuran	C3208160	674.9	C_6_H_8_O	157.998	1.3211	-
52	2-Methylbutanal	C96173	663.8	C_5_H_10_O	155.072	1.40387	-
53	3-Methylbutanal	C590863	650.6	C_5_H_10_O	151.597	1.4118	Dimers
54	1-Butanol	C71363	656.2	C_4_H_10_O	153.06	1.37552	-
55	3-Methylbutanal	C590863	645.1	C_5_H_10_O	150.134	1.20319	Monomers
56	2-Butanone	C78933	591.7	C_4_H_8_O	136.053	1.24854	-
57	2-Methyl propanal	C78842	555.1	C_4_H_8_O	126.36	1.28596	-
58	2-Propanol	C67630	565.4	C_3_H_8_O	129.104	1.22814	-
59	2,3-Butanedione	C431038	582.1	C_4_H_6_O_2_	133.493	1.18732	-
60	Acetic acid	C64197	565.4	C_2_H_4_O_2_	129.104	1.16918	-
61	2-Propanone	C67641	502.4	C_3_H_6_O	112.462	1.11816	-
62	Ethanol	C64175	476.1	C_2_H_6_O	105.513	1.12723	-
63	Methyl acetate	C79209	550.9	C_3_H_6_O_2_	125.263	1.20319	-
64	Toluene	C108883	772.7	C_7_H_8_	195.852	1.01046	-
65	Pentanal	C110623	693.6	C_5_H_10_O	163.667	1.18165	-

**Table 2:** Area of Curcuma volatile oil.

No	Compounds	Molecular Formula	Comment	[+] EZ-1	[+] EZ-2	[+] EZ-3
1	gamma-Decalactone	C_10_H_18_O_2_	-	2912.16	3431.98	3369.21
2	Methyl decanoate	C_11_H_22_O_2_	-	3341.36	3892.70	4013.00
3	(E,E)-2,4-Decadienal	C_10_H_16_O	-	2973.06	3282.99	3434.15
4	Decanoic acid	C_10_H_20_O_2_	-	547.35	633.02	734.73
5	Eugenol	C_10_H_12_O_2_	-	1281.77	1241.07	1233.21
6	2-Decanone	C_10_H_20_O	Monomers	6027.45	6303.08	6420.79
7	2-Decanone	C_10_H_20_O	Dimers	5086.61	5864.29	5719.93
8	alpha-Terpineol	C_10_H_18_O	-	11,283.16	11,913.89	11,295.72
9	Diethyl succinate	C_8_H_14_O_4_	-	1309.52	1346.32	1390.25
10	Citronellol	C_10_H_20_O	-	1096.75	1081.54	1118.81
11	Linalool	C_10_H_18_O	-	9174.36	8964.94	9084.55
12	Ethyl heptanoate	C_9_H_18_O_2_	-	8358.19	8392.94	8400.00
13	2-Nonanone	C_9_H_18_O	-	17,585.94	17,143.82	17,310.05
14	Linalool oxide	C_10_H_18_O_2_	Monomers	4690.67	4701.49	4740.07
15	Linalool oxide	C_10_H_18_O_2_	Dimers	1377.23	1291.99	1354.36
16	1,8-Cineole	C_10_H_18_O	-	12,249.16	12,122.78	12,239.11
17	beta-Ocimene	C_10_H_16_	-	1694.48	1418.96	1385.90
18	Benzeneacetaldehyde	C_8_H_8_O	-	907.73	843.63	960.16
19	Limonene	C_10_H_16_	-	1129.65	1087.89	1100.73
20	alpha-Terpinene	C_10_H_16_	-	918.33	753.93	883.22
21	6-Methyl-5-hepten-2-one	C_8_H_14_O	-	2011.42	1931.01	1962.76
22	beta-Pinene	C_10_H_16_	Monomers	2522.96	2519.12	2598.67
23	beta-Pinene	C_10_H_16_	Dimers	5057.77	4934.82	4995.13
24	Camphene	C_10_H_16_	-	2191.88	1963.45	1998.16
25	alpha-Pinene	C_10_H_16_	Monomers	1366.07	1343.20	1351.68
26	alpha-Pinene	C_10_H_16_	Dimers	3062.82	2762.18	2848.96
27	2-Ethylhexanol	C_8_H_18_O	-	570.80	651.25	626.41
28	Ethyl hexanoate	C_8_H_16_O_2_	-	6863.47	6978.71	7043.41
29	2-Octanone	C_8_H_16_O	-	2915.13	2853.83	2942.25
30	Benzaldehyde	C_7_H_6_O	Monomers	221.68	219.65	238.90
31	Benzaldehyde	C_7_H_6_O	Dimers	473.44	415.37	483.78
32	Ethyl pentanoate	C_7_H_14_O_2_	-	20,873.63	20,994.98	20,390.77
33	2-Heptanone	C_7_H_14_O	-	7475.55	7083.04	7137.75
34	2-Furanmethanol	C_5_H_6_O_2_	-	388.76	373.74	385.81
35	2-Acetylfuran	C_6_H_6_O_2_	-	235.13	147.71	235.75
36	2,5-Dimethylthiophene	C_6_H_8_S	-	373.28	428.12	347.52
37	Furfural	C_5_H_4_O_2_	Monomers	179.78	171.41	169.89
38	Furfural	C_5_H_4_O_2_	Dimers	301.64	223.43	233.77
39	2-Methylbutanoic acid	C_5_H_10_O_2_	-	296.69	266.89	276.09
40	2-Hexanol	C_6_H_14_O	-	7430.20	7138.66	7364.58
41	2-Hexanone	C_6_H_12_O	-	453.71	389.56	391.78
42	2,3-Butanediol	C_4_H_10_O_2_	-	847.31	685.55	654.67
43	1-Pentanol	C_5_H_12_O	-	120.72	108.34	108.60
44	Acetal	C_6_H_14_O_2_	-	113.23	112.71	135.34
45	(E)-2-Pentenal	C_5_H_8_O	-	79.52	61.97	58.52
46	3-Hydroxy-2-butanone	C_4_H_8_O_2_	-	467.65	377.73	358.13
47	2,5-Dimethylfuran	C_6_H_8_O	-	45.71	46.97	51.40
48	Methyl butanoate	C_5_H_10_O_2_	-	74.38	79.05	70.13
49	Ethyl propanoate	C_5_H_10_O_2_	-	1540.94	1377.37	1423.45
50	2-Pentanone	C_5_H_10_O	-	113.31	125.79	49.42
51	2-Ethylfuran	C_6_H_8_O	-	225.15	186.35	177.51
52	2-Methylbutanal	C_5_H_10_O	-	744.68	675.02	583.69
53	3-Methylbutanal	C_5_H_10_O	Dimers	788.51	732.97	689.25
54	1-Butanol	C_4_H_10_O	-	398.41	379.24	285.82
55	3-Methylbutanal	C_5_H_10_O	Monomers	299.34	222.36	229.26
56	2-Butanone	C_4_H_8_O	-	253.22	227.04	159.78
57	2-Methyl propanal	C_4_H_8_O	-	161.44	153.49	88.17
58	2-Propanol	C_3_H_8_O	-	1192.77	1087.52	1045.79
59	2,3-Butanedione	C_4_H_6_O_2_	-	391.70	358.15	347.32
60	Acetic acid	C_2_H_4_O_2_	-	718.70	654.59	652.46
61	2-Propanone	C_3_H_6_O	-	9466.58	8838.34	8918.75
62	Ethanol	C_2_H_6_O	-	2438.91	2048.25	2256.04
63	Methyl acetate	C_3_H_6_O_2_	-	364.86	337.56	295.63
64	Toluene	C_7_H_8_	-	498.55	426.60	460.66
65	Pentanal	C_5_H_10_O	-	94.88	126.62	106.95

## Data Availability

Not applicable.

## Sample Availability

Samples of the compounds are not available from the authors.
